# Realigning the provider payment system for primary health care: a pilot study in a rural county of Zhejiang Province, China

**DOI:** 10.1017/S1463423620000444

**Published:** 2020-10-09

**Authors:** Xiaoying Pu, Ting Huang, Xiaohe Wang, Yaming Gu

**Affiliations:** 1 School of Medicine, Hangzhou Normal University, Hangzhou, China; 2 Shengzhou Traditional Chinese Medicine Hospital, Shaoxing, China; 3 Division of Health Reform, Health Commission of Zhejiang Province, Hangzhou, China

**Keywords:** China, pay-for-performance, primary health care, provider payment, resource-based relative value scale

## Abstract

**Aim:**

This work aimed to evaluate a pre/post-reform pilot study from 2015 to 2018 in a rural county of Zhejiang Province, China to realign the provider payment system for primary health care (PHC).

**Methods:**

Data were extracted from the National Health Financial Annual Reports for the 21 township health centers (THCs) in Shengzhou County. An information system was designed for the reform. Differences among independent groups were assessed using *Kruskal–Wallis H*-test. *Dunn’s post hoc* test was used for multiple comparisons. Differences between paired groups were tested by *Wilcoxon* signed-rank test. Two-tailed *P* < 0.05 indicated statistical significance. Data were processed and analyzed using R 3.6.1 for Windows.

**Findings:**

First, payments to THCs shifted from a “soft budget” to a mixed system of line-item input-based and categorized output-based payments, accounting for 17.54% and 82.46%, respectively, of total revenue in 2017. Second, providers were more motivated to deliver services after the reform; total volumes increased by 27.80%, 19.22%, and 30.31% for inpatient visits, outpatient visits, and the National Essential Public Health Services Package (NEPHSP), respectively. Third, NEPHSP payments were shifted from capitation to resource-based relative value scale (RBRVS) payments, resulting in a change in the NEPHSP subsidy from 36.41 to 67.35 per capita among the 21 THCs in 2017. Fourth, incentive merit pay to primary health physicians accounted for 38.40% of total salary, and the average salary increased by 32.74%, with a 32.45% increase in working intensity. A small proportion of penalties for unqualified products and pay-for-performance rewards were blended with the payments. The reform should be modified to motivate providers in remote areas.

**Conclusion:**

In the context of a profit-driven, hospital-centered system, add-on payments – including categorized output-based payments to THCs and incentive merit pay to primary care physicians (PCPs) – are probably worth pursuing to achieve more active and output/outcome-based PHC in China.

## Background

Appropriate primary health care (PHC) is essential to an efficient and equitable healthcare system, as well as being the best way of meeting challenges such as a rapidly aging society and an increasing burden of noncommunicable diseases (World Health Organization, 2018; Kendall *et al.*, 2019). In China, the PHC system is divided into urban and rural components, which are organized differently but perform the same function, namely, generalist clinical care and basic public health services (Li *et al.*, 2017). In urban areas, the system is composed of community health centers (over 90% publicly owned) and community health stations (about 70% publicly owned). In rural areas, it is composed of township health centers (THCs; almost all publicly owned) and village clinics (about 60% publicly owned) (National Health and Family Planning Commission of the People’s Republic of China, 2017). However, China’s recent attempts to redirect patients to PHC had limited effects on capability, efficiency, and quality (Li *et al.*, 2017; Ta *et al.*, 2020). It is widely assumed that payment systems and incentives are influential in steering the provision of PHC (Eggleston and Hsieh, 2004; Hung *et al.*, 2013; OECD, 2016). Most primary care physicians (PCPs) in China are employed by public primary health institutions (PHIs), and they are mainly paid on a salary basis (World Bank, 2005; Ma *et al.*, 2019). Therefore, both the facility (payments to PHIs) and individual (PCPs’ salary) levels should be explored when shifting passive budgeting payments toward strategic purchasing. Based on an analysis of the major challenges, we previously suggested a reform framework including the pattern of governance and payments to PHIs and employed physicians (Pu *et al.*, 2019).

**Figure 1.**
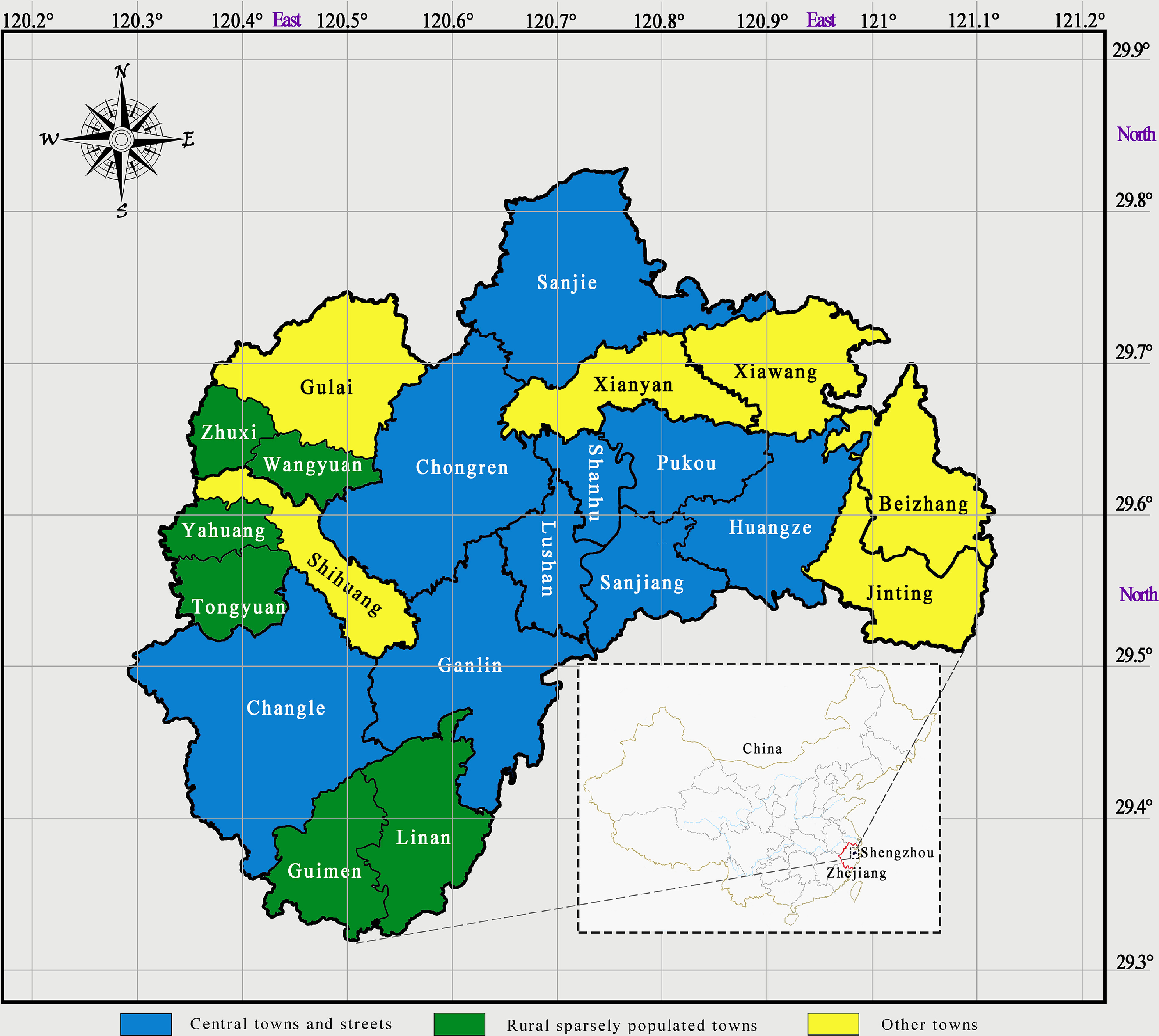
Township health centers’ location in Shengzhou County.

In this work, we specified and examined the framework using Shengzhou County, 1 of the 4 pilot areas among 90 counties in Zhejiang Province, China, as an example. The county contains 21 townships, each of which has a THC (locations are shown in Figure 1). In 2017, urban disposable income per capita was 52 039 Yuan (7709 US dollars), and rural net income per capita was 26 944 Yuan (3992 US dollars) (Zhejiang Bureau of Statistics, 2018). The county had a good health information technology (IT) basis for the pilot reform; it was one of the field study sites for the national digital health key technology and regional demonstration research initiative (Key Project of the National Science and Technology Support Plan) started in 2011, and for the provincial health IT pilot study of family physician service and two-way referral started in 2015. The regional health IT platform has been built, and 18 sets of application software including hospital information systems (HISs) and electronic health records (EHRs) have been put into use.

The pilot was launched in October 2015. The main aspects of the reform are summarized in Table 1. In 2015 and 2016, the focus was on policy design, simulation measurement, and IT construction. After 2 years in practice (2017 and 2018), the Zhejiang Provincial Department of Finance (ZPDF) and Zhejiang Provincial Health Commission (ZPHC) jointly published a document to promote the pilot program (ZPDF and ZPHC, 2017). In collaboration with these two departments, we performed this study to describe the detailed framework of the reform, the changes that have taken place, and the implications for policy and future research.

**Table 1.** The main courses of pilot reform in Shengzhou county, 2015–2018

Date	Contents
15 October 2010	Earmarked funding of 13.5 million Yuan (about 2 million US dollars) was allocated to Shengzhou County by the Zhejiang Provincial Department of Finance.
24 December 2015	A budget of 8.68 million Yuan (about 1.28 million US dollars) for the information system, including data integration and comprehensive evaluation, was approved by the Financial Bureau of Shengzhou County. The project was divided into two phases (phase I: 5.75 million Yuan and phase II: 2.93 million Yuan).
26 April 2016	Information system (phase I) was bid as 5.45 million Yuan (~ 80,700 US dollars).
7 July 2016	An action plan for the pilot reform was issued by the Government of Shengzhou County (Shengzhengban [2016] No. 85).
29 December 2016	Many implementing regulations were released by the Financial Bureau of Shengzhou County and Health Bureau of Shengzhou County, such as standard work equivalent for 36 PHC services in NEPHSP, a weighting index to compensate for variation in different township health centers, and many calculation formulas (Shengwei [2016] No. 174, 175, 176).
23 January 2017	Information system (phase I) was put into use.
20 February 2017	A scheme of comprehensive evaluation was issued by the Health Bureau of Shengzhou County and Financial Bureau of Shengzhou County (Shengwei [2017] No. 10).
7 September 2017	A scheme was issued to optimize performance-based internal remuneration among township health centers (Shengzhengban [2017] No. 123).
11 October 2017	Information system (phase II) was bid as 2.68 million Yuan (~397 000 US dollars).
30 October 2017	A document comprehensively promoting the pilot program was issued by the Zhejiang Provincial Department of Finance and Zhejiang Provincial Health Commission (Zhecaishe [2017] No. 63).
29 November 2017	Information system (phase II) was put into use.
26 February 2018	Earmarked funding of 2.06 million Yuan (~305 000 US dollars) was allocated to Shengzhou County by the Zhejiang Provincial Department of Finance.
10 May 2018	The optimized scheme of realigning the provider payment system for primary health care was issued by the Health Bureau of Shengzhou County and Financial Bureau of Shengzhou County (Shengwei [2018] No. 58).

## Methods

### Reform framework for Shengzhou county

The Shengzhou payment reform aimed to shift the passive budgeting payments toward strategic purchasing (Pu *et al.*, 2019). The county government issued the reform policy in July 2016 (Table 1). The reimbursement framework consisted of two levels. At the facility level, a mixed system of input-based (line-item budget) and categorized output-based payments was launched to meet balanced objectives of equity and efficiency. At the individual level, a basic salary plus a bonus based on performance was given to incentivize PCPs.

#### Input-based payments to THCs

Line-item budgets were used to subsidize the 21 THCs from the supply side. These included five main items: (1) infrastructure, medical equipment, and IT expenditure in accordance with the regional health plans; (2) health personnel recruitment and training; (3) basic salary for each employee based on the 2016 standard and the headcount quota approved by the government; (4) a 60% share borne by the employer for a variety of social security insurances (endowment insurance, medical insurance, occupational annuity, housing accumulation fund, etc.) based on the headcount quota; and (5) extra budgets to deliver health services in remote areas. These five items were further divided into two components, technical and professional, with reference to resource-based relative value scale (RBRVS) payments (Hsiao and Becker, 1989; Lam and Medverd, 2013). The technical component covered budgets attributed to the facility, including (1), (2), and some facility costs. It was budgeted according to needs. The professional component was the payment directly received by PCPs to pay for services including (3), (4), and remote area allowances in (5). It was budgeted annually and allocated monthly. With decentralization in allocating resources, all these line-item budgets were based on local conditions and financed by the county. Fiscal transfer payment from the province to the county was via large block grants weighted by a distribution formula named *Yinsufa* by the ZPDF (Zhecaiyu [2011] No. 43, unpublished). Prior to 2011, these block grants were made as special transfers with some earmarking.

#### Output-based payments to THCs

**Figure 2.**
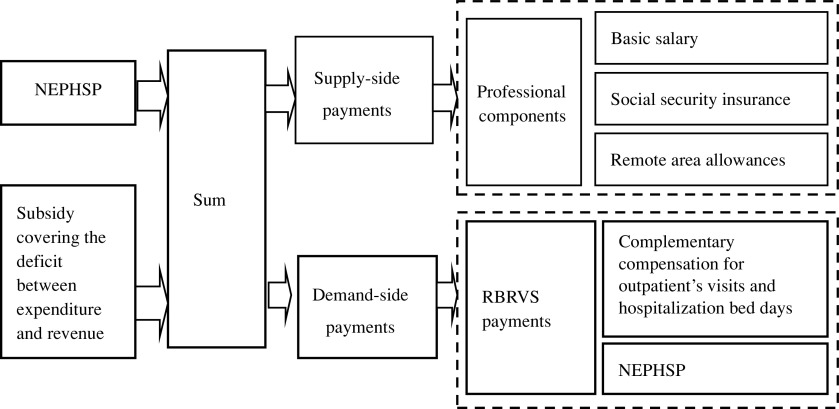
Funding structure of RBRVS reform. Notes: NEPHSP = National Essential Public Health Services Package; RBRVS = resource-based relative value scale.

Payments were categorized according to services provided and sources of funding. The categories included the following:

1. *Projected expenditure financed by budgets*. Major public health service programs, public health emergency response, and family planning technical services were financed by governments and were often managed as projects. The services were paid in the form of the stipulated standard when it existed. Otherwise, they were paid by negotiation beforehand, or the costs were settled after delivery.
2. *RBRVS payment by budgets*. The RBRVS is popularly used to describe, quantify, and reimburse physician services (Hsiao and Becker, 1989; Lam and Medverd, 2013; Lee and Jeong, 2018). It was used to assign the National Essential Public Health Services Package (NEPHSP) and complementary compensation for outpatient visits and hospitalization bed days to quantify the relative work and cost, and to establish appropriate payment. The relative value (RV) of work, derived from magnitude estimation, can be used to represent the workload of each service. The funding structure of the reform is shown in Figure 2. We first integrated the NEPHSP budget and special subsidies covering the deficit between expenditure and revenue into a sum. This sum was divided into professional and activity-based components. The former was derived from input-based payments to PHIs as described above, including basic salary, social security insurance, and remote area allowances. The activity-based component was paid by the RBRVS. RV was used to quantify the NEPHSP, outpatient visits, and hospitalization bed days by comparing their workload with a standard clinic visit (Yin *et al.*, 2015; Pu *et al.*, 2019). To prevent over-consumption on the part of providers, as RBRVS is an activity-based payment, volume thresholds were introduced to limit spending increases according to service standards (OECD, 2016).

The actual reimbursement amount of RBRVS in each THC can be calculated as follows:
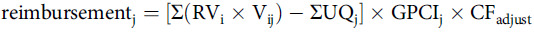



Here, RV_*i*_ is the RV of each PHC service; these RVs are shown in the Appendix, including standard requirements, data source, statistical definition, and quality control (Shengwei [2016] No. 174). *V*_*ij*_ is the corresponding volume in each THC. *V*_*ij*_ is multiplied by RV_*i*_, and these figures are added together to yield the total RV_*j*_ for each THC. ∑UQ_*j*_ is the total of the unqualified RVs in each THC. Spot checks for service quality were conducted twice a year, and the unqualified part was rejected in proportion following the method of sample expansion to the population. GPCI_*j*_ is the geographic practice cost index of each THC, which is decided according to geographical location and the PHIs’ capacities and running costs. The 21 THCs in Shengzhou County were divided into 3 categories (Figure 1); the GPCIs of the 10 central towns, 6 rural sparsely populated towns, and the remaining 5 THCs were set to 1.0, 1.5, and 1.2, respectively. CF_adjust_ is the conversion factor after adjustment that converts RV into Yuan as follows: CF_adjust_= (CF_pre_ × Total RV)/(∑(RV_*j*_ × GPCI_j_), where CF_pre_ was set to 11.62 Yuan on the basis of historical data and budgets, and RV_*j*_ is the total RV of each THC.

(3) *Mixed payments for outpatient and inpatient services financed by medical insurance and co-payment*. Although fee-for-service (FFS) remained the predominant payment method for outpatient and inpatient services, the county has been moving toward a mixed payment scheme. A global budget control was launched in 2012, setting an annual reimbursement cap for basic medical insurance. The cap was to some extent arbitrary and was based on historical revenues. As the general practitioner system was at an early stage, a blended pilot payment for outpatient services was used, including capitation for qualified contracted residents and FFS for other residents in one THC (Huangze) in June 2019. The pilot study was extended to three other THCs (Ganlin, Gulai, and Beizhang). Unfortunately, the data did not allow us to analyze the overall efficiency and results in this study.

#### Payments to PCPs

The finance and health bureaus of Shengzhou County issued a document to optimize performance-based internal remuneration in September 2017 (Table 1). The main measures included the following:

1. *Salary composition*. The salary consisted of basic salary and merit pay based on performance. Basic salary was relatively stable and was related to individual factors: seniority, qualification, and professional title. Merit pay was dynamically adjusted and included basic and incentive merit pay. The former was related to the position being appointed and was allocated monthly in advance; the latter was paid after twice-yearly performance evaluation, including performance appraisal award, allowance for special post operations, and allowance for unclaimed annual leave.
2. *Total amount of merit pay*. This was regulated by the base-plus-increase pattern for each THC. Basic merit pay was set according to the level of the previous year; incentive merit pay included annual dynamic rises, a year-end performance bonus set by administrative departments in Shengzhou County (50% of the funding comes from institution expenditure and 50% from extra fiscal budgets), and 50% of the final account surplus.
3. *Salary distribution among employees*. THCs were endowed by the administrative department with administration autonomy so as to stimulate endogenous power and operational vitality. Each THC can decide the proportion of performance-based bonuses, the merit rating method, rewards for outstanding contributors, and the remote area allowance by themselves. However, the authorities reserve the power to regulate equity and efficiency. For example, the highest and lowest merit pay levels are controlled by the government.

### Data collection

All 21 THCs were involved in the reform. As shown in Table 1, many regulations were released during 2015 and 2016. Thus, we selected data for 2015 and 2016 as the pre-reform baseline, while the 2017 and 2018 data were used to represent the post-reform situation. The pre-reform results were calculated using a simulation method. Three datasets were extracted as follows. (1) Basic information, income, and expenditure of THCs were obtained from the National Health Financial Annual Report of 2015–2018 (table for general statement of income and expenditure, table for lists of revenue and expenditure from government subsidies, and table for basic figures and financial analysis). (2) RBRVS payments to the NEPHSP and complementary compensation for outpatient visits and hospitalization bed days were obtained from the IT platform designed for this reform. (3) Employee salaries were obtained from the personnel and salary management system. However, we only obtained anonymized results after analysis because of privacy considerations.

### IT system

The budget to set up the Shengzhou County IT system in December 2015, including data integration and comprehensive evaluation, was 8.68 million Yuan (about 1.28 million US dollars). The project was divided into two phases (phase I: 5.75 million Yuan and phase II: 2.93 million Yuan), which were launched in January and December 2017, respectively (Table 1). The IT system consisted of four main parts. First, a data integration platform was set up to collect complex, heterogeneous information (e.g., HISs, EHRs, immunization records, maternal and child health management data, severe mental disorder management, etc.). Second, the county established a quality control management system covering data source, data collection, data processing, cost calculation, and appeal feedback. Third, based on the data integration platform and assessment index database, performance assessment and provider payment systems were established at institutional and individual levels. Fourth, an RBRVS payment system was set up according to the funding structure of the reform (Figure 2) and the reimbursement formula.

### Statistical analysis

Descriptive statistics (frequencies, percentages, line charts, box plots, etc.) were used to analyze the data. The differences in total salaries among independent groups (central towns and streets, sparsely populated rural towns, and others) were assessed by *Kruskal–Wallis* H-test. Dunn’s post hoc test was used for multiple comparisons of average salary. Differences in annual income between permanent and temporary employees were analyzed by *Wilcoxon* signed-rank test. Two-tailed *P* < 0.05 was considered to indicate statistical significance. Data were processed and analyzed using R 3.6.1 for Windows.

## Results

### Basic information

The management systems of the 21 THCs were almost unchanged during 2015 and 2018, with the exception of the provider payment system. Notably, the number of employees decreased by 1.92% (from 1510.5 to 1481.5) after the reform, but personnel structures improved to varying degrees (Table 2). The percentages of permanent employees and health professionals increased by 3.88% (from 68.75% to 72.63%) and 4.62% (from 84.61% to 89.23%). An additional 59 beds (from 595 to 654) were made available in these 21 THCs after the reform. Outpatient visits increased from 2.71 million to 3.23 million (growth rate: 19.19%), and inpatient visits increased from 15 650 to 20 000 (growth rate: 27.80%). The average salary of each employee increased by 32.75% from 87 520 to 116 180 Yuan (without adjustment for inflation).

**Table 2.** Pre- and post-reform changes

Items	Pre-reform			Post-reform		
	2015 (%)	2016 (%)	Mean (%)	2017 (%)	2018 (%)	Mean (%)
Total employees	1458 (100%)	1563 (100%)	1510.5 (100%)	1490 (100%)	1473 (100%)	1481.5 (100%)
#Permanent ones	1015 (69.62%)	1062 (67.95%)	1038.5 (68.75%)	1062 (71.28%)	1090 (74.00%)	1076 (72.63%)
#Health professionals	1264 (86.69%)	1292 (82.66%)	1278 (84.61%)	1321 (88.66%)	1323 (89.82%)	1322 (89.23%)
Total beds opened	588	602	595	660	648	654
Total outpatients (million)	2.64	2.77	2.71	3.17	3.28	3.23
Total inpatients (thousand)	13.4	17.9	15.65	19.4	20.6	20
Total revenue	360.80 (100%)	400.47 (100%)	380.64 (100%)	479.62 (100%)	528.79 (100%)	504.21 (100%)
#Financial subsidy	113.26 (31.39%)	114.81 (28.67%)	114.04 (29.96%)	153.26 (31.95%)	172.90 (32.70%)	163.08 (32.34%)
#Medical income	237.19 (65.74%)	277.88 (69.39%)	257.54 (67.66%)	301.14 (62.79%)	345.30 (65.30%)	323.22 (64.10%)
Total expenditure	364.13	415.76	389.95	474.35	510.46	492.41
Net income (surplus rate %)	−3.34 (−0.93%)	−15.29 (−3.82%)	−9.32 (−2.45%)	5.27 (1.10%)	18.33 (3.47%)	11.80 (2.34%)
Average salary for employees (1000 Yuan)	78.55	96.10	87.52	102.34	129.67	116.18

Notes: Total revenue, expenditure, net income, and average salary for permanent employees were not adjusted for inflation.

### Payments to THCs

**Figure 3.**
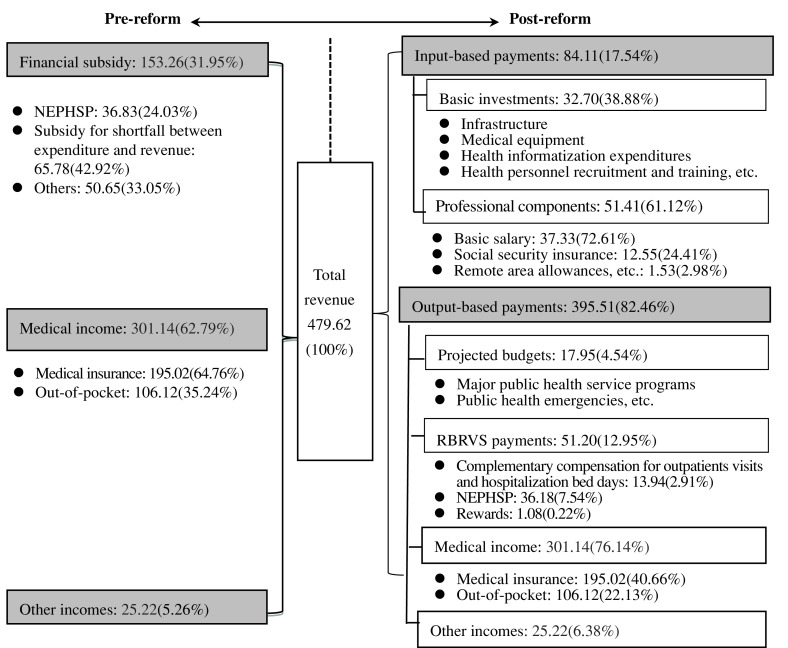
Payments to township healthcare centers in 2017 (million Yuan). Notes: NEPHSP = National Essential Public Health Services Package; RBRVS = resource-based relative value scale.

Total revenue increased by 32.46% from 380.64 million Yuan pre-reform to 504.21 million Yuan post-reform (Table 2). This 123.57 million Yuan increase mainly came from medical income (65.68 million Yuan, 53.15%) and financial subsidies (49.04 million Yuan, 39.69%). Moreover, the balance of payments improved, from a loss of 9.32 million Yuan (loss rate: 2.45%) to a surplus of 11.80 million Yuan (surplus rate: 2.34%). We used 2017 data to illustrate the differences between payments before and after reform (Figure 3). Prior to reform, total revenue was composed of financial subsidies (153.26 million Yuan, 31.95%), medical income (301.14 million Yuan, 62.79%), and other incomes (25.22 million Yuan, 5.26%). To solve the problem of low efficiency, which was mainly caused by lump-sum payments for the NEPHSP (36.83 million Yuan, 31.95% of financial subsidy) and a “soft budget” for the deficit after the zero make-up policy (65.78 million Yuan, 42.92% of financial subsidy), we reformed these payments (Figure 2, right side). After the reform, input- and output-based payments accounted for 17.54% (84.11 million Yuan) and 82.46% (395.51 million Yuan), respectively, of total revenue in 2017. (Figure 3). Input-based payments were composed of basic investments (32.70 million Yuan, 38.88%) and professional components (51.41 million Yuan, 61.12%). Output-based payments were composed of medical income (301.14 million Yuan, 76.14%), RBRVS payments for the NEPHSP and complementary compensation for outpatient visits and hospitalization bed days (51.20 million Yuan, 12.95%), projected budgets such as major public health service programs (17.95 million Yuan, 4.54%), and other incomes (25.22 million Yuan, 6.38%).

### RBRVS payment reform

The results for the reformed RBRVS funding structure (Figure 2) are shown in Table 3. The pre-reform data were calculated using a simulation method. First, the total fund going through the reform accounted for about two-thirds of financial subsidies (Table 3). The proportions were 66.95% and 64.11% in 2017 and 2018, respectively, with a small difference between pre- and post-reform (67.38% vs. 65.45%). Second, the percentage of professional components contributing to the total fund was relatively low (38.35%) in 2015 (Table 3), with the intention to have higher activity-based motivation at the simulation stage. However, many managers of THCs expressed a view that this should be gradually reduced in order to motivate active PCP participation in early-stage reform. Thus, the percentage of professional components was increased to 51.51% in 2016 for the simulation stage. After formal implementation, this gradually decreased to 50.10% and 45.13% in 2017 and 2018, respectively (Table 3). Third, RBRVS payments grew by 35.15% compared with pre-reform (55.91 million Yuan vs. 41.37 million Yuan). Among the RBRVS payments, the NEPHSP accounted for 70.66% and 71.59%, respectively, in 2017 and 2018 (Table 3). The total RV of the NEPHSP also increased by 30.31% from 2.54 million points pre-reform to 3.31 million points post-reform. The reward for RBRVS payments was very small (1.02 million Yuan, 1.82% of total), but it showed a good trend for blended activity-based payments and pay-for-performance. Fourth, a small proportion of unqualified services were not paid; 0.24 and 0.23 million points (RV) in 2017 and 2018, accounting for nearly 5% of the total RV. This was conductive to enhancing THC quality awareness. Fifth, each employee’s work intensity increased year by year. For example, RV points per employee were 2273.64, 2434.75, 2959.73, and 3285.81 in 2015, 2016, 2017, and 2018, respectively (Table 3). This corresponded to a 32.45% increase after reform (3121.84 points post-reform vs. 2357 points pre-reform).

**Table 3.** RBRVS payment changes between pre- and post-reform (million Yuan)

Items	Pre-reform*			Post-reform		
	2015	2016	Mean	2017	2018	Mean
NEPHSP (1)	31.38	34.12	32.75	36.83	43.40	40.12
Subsidy covering the deficit between expenditure and revenue (2)	31.10	57.08	44.09	65.78	67.45	66.62
Sum of reform (3) = (1)+(2) = (6)+(8)	62.48	91.20	76.84	102.61	110.85	106.73
Financial subsidy (4)	113.26	114.81	114.04	153.26	172.90	163.08
# Sum of reform as % (5) = (3)/(4)*100%	55.17%	79.44%	67.38%	66.95%	64.11%	65.45%
Professional components (6)	23.96	46.98	35.47	51.41	50.23	50.82
#As % of sum of reform (7) = (6)/(3)*100%	38.35%	51.51%	46.16%	50.10	45.13	47.13
RBRVS payments (8) = (9)+(11)+(12)	38.52 (100%)	44.22 (100%)	41.37 (100%)	51.20 (100%)	60.62 (100%)	55.91 (100%)
# NEPHSP (9)	28.11 (72.98%)	31.14 (70.42%)	29.625 (71.61%)	36.18 (70.66%)	43.40 (71.59%)	39.79 (71.17%)
NEPHSP per capita (Yuan) (10)	38.34	42.63	40.48	49.54	59.51	54.52
#Complementary compensation for medical service (11)	10.41 (27.02%)	13.08 (29.58%)	11.745 (28.39%)	13.94 (27.23%)	16.27 (26.84%)	15.11 (27.02%)
#Rewards (%) (12)	0 (0%)	0 (0%)	0 (0%)	1.08 (2.11%)	0.95 (1.57%)	1.02 (1.82%)
Total RV before quality control(million points) (13)	3.31	3.81	3.56	4.65	5.07	4.86
Total unqualified RV(million points) (14)	–	–	–	0.24	0.23	0.24
Total RV after quality control (million points) (15) = (13)−(12) = (16)+(17)	3.31 (100%)	3.81 (100%)	3.56 (100%)	4.41 (100%)	4.84 (100%)	4.62 (100%)
#NEPHSP (16)	2.39 (72.10%)	2.68 (70.42%)	2.54 (71.20%)	3.17 (71.88%)	3.44 (71.07%)	3.31 (71.54%)
#Medical service(17)	0.92 (27.90%)	1.13 (29.58%)	1.03 (28.80%)	1.24 (28.12%)	1.40 (28.93%)	1.32 (28.46%)
CF of RBRVS (Yuan/point) (18)	11.62	11.62	11.62	11.61	12.52	12.10
RBRVS each employee (points/per year) (19)	2273.64	2434.75	2357.00	2959.73	3285.81	3121.84

Notes: The results pre-reform were calculated using the simulation method. CF = conversion factor; NEPHSP = National Essential Public Health Services Package; RBRVS = resource-based relative value scale; RV = relative value.

### NEPHSP payments per capita and professional subsidy per employee

**Figure 4.**
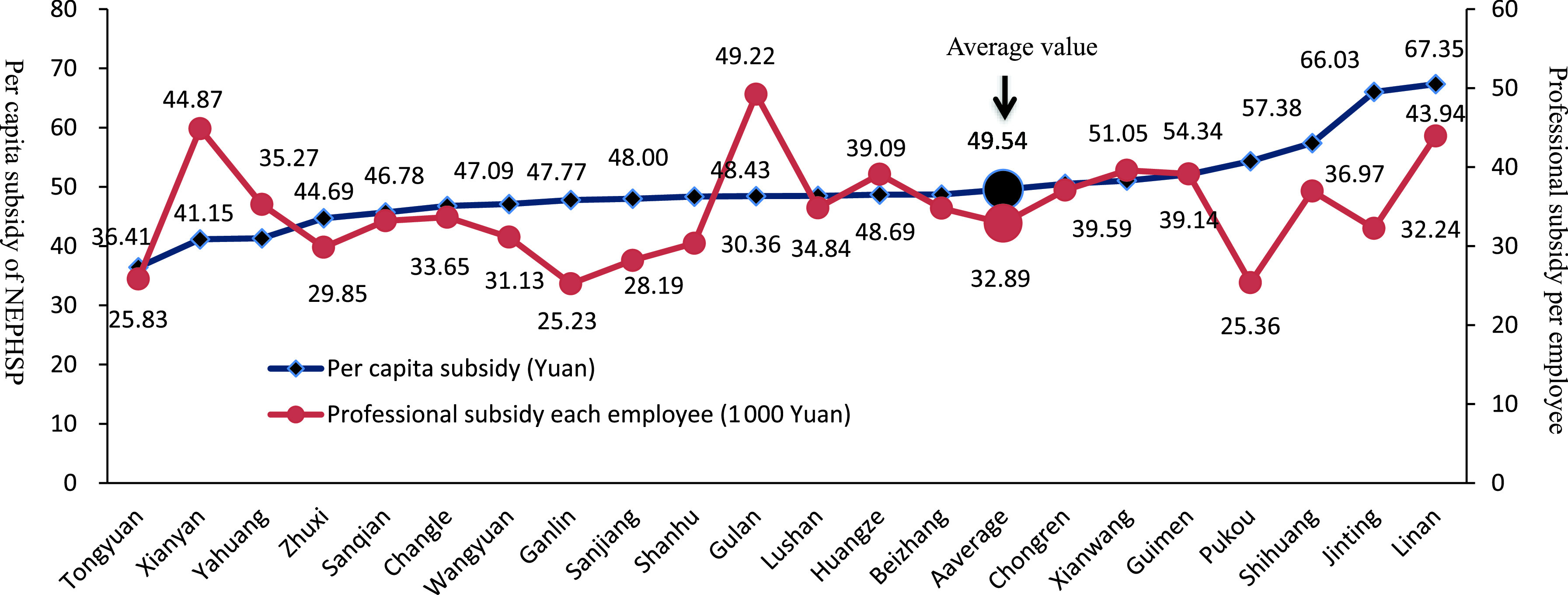
Per capita subsidy of NEPHSP and professional subsidy for each employee for 21 township healthcare centers in 2017. Notes: NEPHSP = National Essential Public Health Services Package; the two large circles represent the averages.

The variation in NEPHSP subsidies per capita among the 21 THCs was small before the reform. Figure 4 illustrates the change after the RBRVS payment reform. NEPHSP subsidies per capita ranged from 36.41 Yuan to 67.35 Yuan per capita, with an average level of 49.54 Yuan among the 21 THCs in 2017. THCs were encouraged to provide more services under the volume thresholds; however, many aspects still need to be improved. For example, there were difficulties for sparsely populated rural THCs. Professional subsidies for each employee were 25 830, 29 850, and 31 130 Yuan for Tongyuan, Zhuxi, and Wangyuan, respectively; these values were below the average of 32 890 Yuan (Figure 4). To balance the fairness incentives, more attention to professional subsidies to employees is required.

### Payments to PCPs

**Figure 5.**
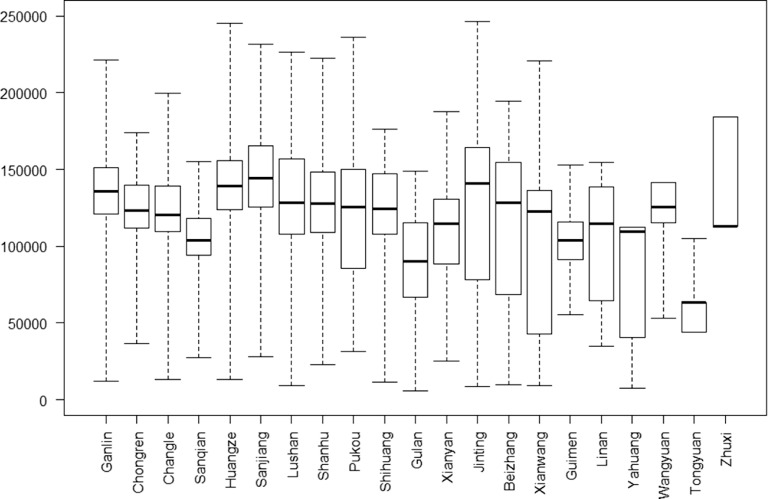
Box plot of annual salary of employee from 21 township healthcare centers in 2017 (Yuan).

The PCP salary composition is shown in Table 4. It consisted of basic salary (20.56%), basic merit pay (41.03%), and incentive merit pay (38.40%) in the 21 THCs of Shengzhou County in 2017. There was little difference in the percentage composition of salary among the three types of THCs. The THCs were given more autonomy to decide salary distribution among employees after the reform (Shengzhengban [2017] No. 123, Table 1). The average salary was 102 340 Yuan among 1490 employees in the 21 THCs. However, it differed greatly among the three types of THCs. The more remote the town, the lower the per capita income. For example, the average salary in central towns and streets was 43.90% more than that in sparsely populated rural towns (106 250 vs. 73 840 Yuan). This difference was statistically significant (*Z* = 12.167, *P* = 0.001) (Table 4). The box plot for annual salary further illustrated the differences among the 21 THCs in 2017 (Figure 5).

**Table 4.** Salary composition of primary care physicians in 2017 (million Yuan)

Category	Townships	Basic salary		Basic merit pay		Incentive merit pay		Total		
		Amount	As % of total	Amount	As % of total	Amount	As % of total	Amount	Employees	Per employee (1000 Yuan)
Central towns and streets	Ganlin	4.65	18.91	9.96	40.50	9.99	40.59	24.60	236	104.25
	Chongren	3.77	21.74	6.61	38.08	6.97	40.18	17.36	169	102.70
	Changle	4.27	23.10	7.20	38.95	7.01	37.95	18.48	166	111.33
	Sanqian	2.97	25.64	5.05	43.56	3.57	30.79	11.59	127	91.29
	Huangze	1.75	18.46	4.28	45.17	3.44	36.37	9.47	83	114.10
	Sanjiang	2.25	18.17	5.31	42.92	4.82	38.91	12.38	110	112.54
	Lushan	1.54	18.12	3.41	40.24	3.53	41.63	8.47	81	104.60
	Shanhu	3.39	22.17	6.16	40.37	5.72	37.46	15.27	133	114.80
	Pukou	1.18	17.11	2.77	40.10	2.95	42.79	6.90	67	102.98
	Average	2.86	20.69	5.64	40.76	5.33	38.55	13.84	130.2	106.25
Other towns	Shihuang	1.33	22.46	2.43	41.15	2.15	36.38	5.91	59	100.19
	Gulan	0.77	23.05	1.38	40.94	1.21	36.01	3.36	47	71.47
	Xianyan	0.65	25.91	1.02	40.66	0.84	33.42	2.52	28	89.90
	Jinting	0.88	14.61	2.63	43.95	2.48	41.44	6.00	56	107.06
	Beizhang	0.60	17.73	1.43	42.39	1.35	39.88	3.38	39	86.73
	Xianwang	0.42	18.85	0.92	41.39	0.88	39.76	2.22	27	82.38
	Average	0.77	19.88	1.64	41.99	1.49	38.13	3.90	42.7	91.37
Rural sparsely populated towns	Guimen	0.29	22.52	0.59	46.06	0.40	31.41	1.28	17	75.44
	Linan	0.31	18.85	0.75	45.59	0.59	35.56	1.65	21	78.72
	Yahuang	0.07	19.73	0.14	37.15	0.16	43.12	0.38	6	63.33
	Wangyuan	0.11	21.51	0.19	36.79	0.22	41.69	0.52	7	74.35
	Tongyuan	0.11	29.28	0.14	37.98	0.12	32.74	0.37	5	74.86
	Zhuxi	0.05	14.30	0.17	47.23	0.14	38.47	0.37	6	61.29
	Average	0.16	20.74	0.33	43.53	0.27	35.73	0.76	10.3	73.84
Total average		1.49	20.57	2.98	41.03	2.79	38.40	7.26	71.0	102.34
*χ*^*2*^		17.161	–	17.544	–	17.532	–	17.544	17.544	14.139
*P*		<0.001	–	<0.001	–	<0.001	–	<0.001	<0.001	0.001*

Notes*: χ2* of Kruskal–Wallis *H* test, *Dunn’s post hoc test showed that the central towns and streets group was significantly higher than the rural sparsely populated towns group (*Z* = 12.167, *P* = 0.001).

## Discussion

This paper describes a payment framework for public THCs and the subsequent internal salary remuneration to PCPs in China. In the pilot study in Shengzhou County, payments to THCs were shifted from a “soft budget” to a mixed system of line-item input-based payments and categorized output-based payments. Here, the term “soft” refers to the lack of enforced financial responsibility and low residual claimant power (Eggleston *et al.*, 2009). The aim was to solve the problem of insufficient government investment and low performance by realigning demand and supply-side incentives (Powell-Jackson *et al.*, 2015; Ma *et al.*, 2019). Line-item budgets are characterized by the allocation of resources to providers, with benefits in terms of accessibility and cost reductions in choosing appropriate providers (Waters *et al.*, 2004). However, the use of input-based payments only may create problems such as low responsiveness and undertreatment (Gosden *et al.*, 2001; Pu *et al.*, 2019). Output-based payments were used to counter these potential disadvantages. Also, it is advisable to determine the balance point between supply-side and demand-side payments according to local conditions in a transitional context. Input- and output-based payments accounted for 17.54% and 82.46%, respectively, of the total revenue in 2017. Another feature of the pilot study was the fiscal transfer payment from the province to the county; there was a shift from earmarked grants to large block grants, which were weighted by a distribution formula (*Yinsufa*) in Zhejiang Province. Yin’s study showed that earmarked subsidies and tax rebates were the most unequal fiscal transfer schemes (Yin, 2008). By contrast, panel data from Zhejiang Province during 1995–2005 indicated that revenue and expenditure decentralization both promoted allocative efficiency (Stefan, 2013).

As the vast majority of OECD countries use blended forms of payments in primary care, the payments in the pilot study provided substantial information, with total revenue to the 21 THCs increasing by 32.46% after the reform (OECD, 2016). There is some evidence that THCs were more motivated to deliver PHC services after the reform. The total volume of services increased by 27.80%, 19.22%, and 30.31% for inpatient visits, outpatient visits, and NEPHSP purchasing, respectively. Average salary per employee increased by 32.74%, with a 32.45% increase in work intensity (RV points per employee). This can be partially attributed to output-based payments, which were categorized according to services provided, and sources of funding for different payment mechanisms were applied to different healthcare services (OECD, 2016). Moreover, output-based payments incentivized providers to deliver more services at the PHC level, consistent with the growing trend toward a greater role for PHC within the whole system (Starfield *et al.*, 2005). This output-based method of payment is of relatively high practical value before dominant FFS has not been reformed in the hospital-centered delivery system in China.

Another change was the governance of THCs after the reform. THC managers had greater autonomy and residual claims, enabling them to keep their costs down, accompanied by greater accountability and competition among THCs. These factors stimulated managers to focus on increasing revenue and reducing expenditure (OECD, 2016). For example, work was more intense after the reform (32.45% increase), but the number of employees decreased by 1.92% with a better structure. Without reform, most THC managers would have applied to recruit staff, because the personnel subsidy was mainly based on the number of employees (Weng, 2012).

Equitable PHC delivery was of interest in this pilot study. For supply-side payments, line-item subsidies were budgeted, including remote area allowances. PHC practitioners in Estonia are paid through a mixed payment system comprising capitation and additional remuneration, including a distance allowance (Dan and Savi, 2015). For demand-side payment, we introduced the GPCI based on THC geographic location, capacity, and condition. An additional budget valued at 3 million Yuan was set up to buffer the risk of overages due to reform (Table 1). With regard to individual payments, basic salary and basic merit pay accounted for 64.27% of total salary in six rural sparsely populated THCs, higher than in the other two types of THCs (61.45% and 61.87%). However, inequality remains a problem in Shengzhou County. The incomes of central THC employees were markedly higher than those of others. Some measures should be intensified, including remote area allowances, professional subsidy for each employee, and GPCI.

The capitation payment to the NEPHSP, combined with salary payments to employees, tended to create incentives for undertreatment and risk selection (Gosden *et al.*, 1999; Liu *et al.*, 2019). RBRVS, an activity-based payment, was introduced to pay the NEPHSP, using volume thresholds to prevent supplier-induced demand, because both over- and undertreatment are unacceptable (Gosden *et al.*, 2001). In this pilot study, providers were motivated to deliver more and better services, for the following reasons. First, the total qualified RV of RBRVS increased by 29.78%, with a 35.15% larger budget for RBRVS after the reform. Second, unqualified RV, accounting for nearly 5% of the total, was not paid via the computer-aided quality control system. Third, a small proportion (1.82% of the total) of pay-for-performance was blended with RBRVS payments. Four, there was a meaningful increase in NEPHSP subsidies from 36.41 to 67.35 Yuan per capita among the 21 THCs in 2017.

The pilot reform was very complex to administer, as it required data systems for collection, measurement, and reimbursement calculations (OECD, 2016; Pu *et al.*, 2019). Although Shengzhou County had good IT infrastructure and received strong support from the provincial authorities, including funding and implementation support, designing the IT system was a very complex process. Thus, it would be practical to divide the system into two or even more stages after scientific design. Moreover, it was operationally helpful for the county to use existing data and reporting requirements as a starting point to reduce administrative costs (OECD, 2016). However, it was also necessary to develop some new systems or modify existing systems. For indicators that could not be included in the IT system during the project, an auxiliary data entry interface was provided as an interim measure. For example, there were 44 performance indicators in 2018; 41 were automatically collected by the IT system, and 3 were manually completed. In addition, the IT system offered monitoring, evaluation, and feedback reporting to the THCs on a systematic basis, thereby encouraging providers to improve quantity and quality.

This study had several limitations. First, it relied on government administrative data, which might contain reporting errors. However, our study primarily used health financial records, which are more reliable because they are approved by the health authorities and randomly audited by relevant departments. Second, as with any quasi-experimental approach, there was the possibility of bias in our estimates of impact, the volume of each service, and the RVs. The study lacked a control group, which influenced its external validity. Third, owing to data limitations, we could not examine the impact of employee attitude. Noneconomic incentives are also important, and issues such as future career development should be of more concern (Zhang *et al.*, 2016; Liu *et al.*, 2019). Finally, our findings were applicable to Shengzhou County, and we are cautious in generalizing to other places.

## Conclusions

Based on a pilot study in Shengzhou County of Zhejiang Province in China, we evaluated the payment framework for public THCs and the subsequent internal salary remuneration to PCPs. Our study showed that providers were more motivated to deliver services after the reform, with increases in total volume, working intensity, and average salary. Under the context of a profit-driven, hospital-centered system, add-on payments (on top of existing payments), including categorized output-based payments to THCs and incentive merit pay to PCPs, are probably worth pursuing to achieve more active and output/outcome-based PHC in China. The framework used in the pilot study should be modified to motivate providers in remote areas. All these findings indicate the usefulness of further shifting passive budgeting payments toward strategic purchasing for PHC in China, despite limitations of the study including the lack of a control group and external validation in other settings.

## Supplementary material

For supplementary material accompanying this paper visit https://doi.org/10.1017/S1463423620000444. click here to view supplementary material
